# Resolution invariant wavelet features of melanoma studied by SVM classifiers

**DOI:** 10.1371/journal.pone.0211318

**Published:** 2019-02-06

**Authors:** Grzegorz Surówka, Maciej Ogorzalek

**Affiliations:** Faculty of Physics, Astronomy and Applied Computer Science, Jagiellonian University, Kraków, Poland; Griffith University, AUSTRALIA

## Abstract

This article refers to the Computer Aided Diagnosis of the melanoma skin cancer. We derive wavelet-based features of melanoma from the dermoscopic images of pigmental skin lesions and apply binary C-SVM classifiers to discriminate malignant melanoma from dysplastic nevus. The aim of this research is to select the most efficient model of the SVM classifier for various image resolutions and to search for the best resolution-invariant wavelet bases. We show AUC as a function of the wavelet number and SVM kernels optimized by the Bayesian search for two independent data sets. Our results are compatible with the previous experiments to discriminate melanoma in dermoscopy images with ensembling and feed-forward neural networks.

## Introduction

Melanoma, the neoplasm of the pigment cells of the skin, is still a challenge both for clinicians and CAD (Computer Aided Diagnosis) specialists. Observations with the naked eye and with instruments, especially with popular optical or even with digital dermoscopes require long-term experience which is hard to achieve not only for general practitioners but also for dermatologists. Diagnosis of mature melanoma moles, due to asymmetry, variety in colors or border irregularity, may not be difficult, which is quite the contrary to early melanoma lesions that lack those indications. Effective treatment of melanoma i.e. a high (>95%) 5 or 10-year survival rate consists in early detection and resection of the malignant skin lesion [1]. When not excited at an early stage, melanoma penetrates deep from epidermis to the skin and finally transfers to the lymph nodes and other internal organs by metastasis. At this stage the mortality rate is extremely high and especially for at least a decade has become a medical problem. This problem refers to all countries but particularly these where melanoma morbidity rate is elevated. Statistics says that women got melanoma moles on the legs and men on the back.

Crucial for improving the patient survival rate is early detection and for that the only reliable method is biopsy. For obvious reasons (surgical complications, ANS-Atypical Nevus Syndrome, economic reasons) excision cannot be a diagnostic tool or standard mass treatment. The key role is precise detection of the tumor.

Clinical diagnosis of early or micro-melanoma is extremely difficult even for experts. A differentiation between pigment and non-pigment skin lesions may be a challenging task but classification of different forms of pigment (melanocytic) lesions can be very complex. From the clinical point of view three forms of pigment lesions can be misdiagnosed: benign nevus, dysplastic (atypical) nevus and melanoma. The dysplastic nevus may be a precursor to malignancy and often exhibits features visually identical or close to melanoma [2].

On that account melanoma CAD systems are very popular and of key importance. They help in the early diagnosis of the skin lesions and support non-invasive methods in dermatology. The CAD methods implement machine learning paradigms and are based on data sets of (histopatology or expert ground truth) known cases. Unfortunately such data sets are sparse and with limited statistics of events and perhaps limited or unknown quality (e.g. image resolution, compression).

Recognition of melanoma with such computer systems can be divided into: i) direct machine learning (ML) to recognize melanoma from its features, and ii) ML of specific skin lesion patterns/structures that are indicative of melanoma (lesion border, blue veil, geometric asymmetry etc.). The main feature types classified in the melanoma recognition systems include color and/or geometry-based features, histogram-like features, wavelet-based features and other less common (e.g. Gabor etc.). Literature on the melanoma CAD is ample and deal with all the steps: accuracy of clinical and histopathological examinations (i.e. the ground truth), dermoscopy and digital acquisition of lesion images, preprocessing (removal of artifacts, filtering), and finally pattern recognition and machine learning of melanoma. The majority of articles refer to dermoscopy and/or digital imaging of melanoma, since spectral, trans-illumination, ultrasonography or tomography systems, although promising, are not mass skin diagnostic systems.

In 2013 Masood et al. [3] described the state of the art of the melanoma classification of dermoscopic images with statistics, plus they compared and discussed the results and conditions that affected the analyzed techniques. Later on we refer to this work to scrutinize our results.

The latest review on computational methods and their applications as well as trends for automatic melanoma diagnosis was published in 2016 [4]. It was focused on feature extraction, feature selection, classification algorithms and evaluation procedures.

Overview of statistics and results from the melanoma CAD and segmentation issues can be also found in [5, 6].

The main achievement of this work is first quantification of the C-SVM classification performance of melanoma (in terms of AUC) over a broad range of wavelet bases and SVM kernels. This article is a continuation of our latest research on wavelet-based features of melanoma, derived from dermoscopy images, and classified with various ML paradigms [7, 8]. Our motivation is driven by a systematic search for resolution invariant features and learning methods contributing to the computer pattern recognition of cutaneous melanoma.

### Motivation

The first and still important approaches to the melanoma detection were segmentation techniques [4–6, 9]. Since dermoscopy images can be taken under different conditions, such techniques can suffer from unstable image illumination, different optical magnification, different skin complexions and presence of artifacts (hairs, reflections, bubbles of immersion fluid etc.). Under such circumstances successful pattern recognition is still possible, but may be limited in performance or strongly sensitive to datasets. Identification methods based on spatial and frequency information found in the skin texture can be a promising alternative.

Application of the wavelet- and wavelet-packet- transform to human skin data was first applied in [10]. Patwardhan et al. [11] studied wavelet packets to decompose the sub-bands of the pigmented skin texture, which proved to derive features sensitive to the class of the dermoscopy image [7, 8, 12] and then be subject to appropriate machine learning methods. Some WPT attempts are also shown here [13–15].

Most of the melanoma wavelet-based classifiers quoted in the literature used only a single wavelet base (usually Daubechies 3 or 4 [11, 16]) to build classification models. In our latest research all popular and well defined wavelets are used. We showed that some selected wavelet bases prove to be more efficient and robust for the machine learning of dermoscopic images than the others. They also keep high classification efficiency in the downgraded image resolutions, that is exhibit resolution invariance.

In [7] this was analyzed in the ensemble of different model types, and in [8] in simple back-propagated neural classifier setups. In this work we specifically study SVM classifiers of the wavelet-based features for the melanoma detection to analyze how wavelet bases can affect the quality of the margin-maximization learning paradigm. The choice of SVM is due to the inherent advantages of the method: i) the ability to generalize well as it maximizes the margin between the classes, ii) no local minima, and iii) flexibility of kernel selection within a unified architecture.

Although in this article we want to study and compare with the previous works and literature the SVM classifiers (our work is the first quantification of the C-SVM classification performance of melanoma over a broad range of wavelet bases and SVM kernels), our primary objective is to contribute to optimal feature extraction methods. We believe that adequate wavelet base(s) representation can both yield high classification efficiency and be robust and efficient in worse or reduced resolution environments. For that objective we study one by one different learning paradigms, SVM included.

Computational (memory, time) aspects of any machine learning experiment (not particularly SVM) are important factors which should be carefully planned. This is not only the algorithm convergence and regularness that matters, but if the developed classifier is to be used e.g. to support medical diagnosis (in CAD this is the ultimate goal), practical aspects play a role. Computer parts and systems constantly evolve. Nowadays personal smartphones with ARM-based processors are widely used as medical diagnostic tools, the melanoma CAD included (e.g. [17], see [18] for a review). Since (usually) ML algorithms for image recognition demonstrate high complexity, and small hand-held devices have limited processing power and memory (and the battery capacity), it is of great importance both to: efficiently probe the parameter space of the solution, and to look for features and learning paradigms that preserve high efficiency also in lower image resolutions. In this work our objective is to select the best wavelet bases in terms of AUC classification performance of the SVM binary classifiers of melanoma/dysplastic lesions and analyze how those wavelet bases perform with the reduced image resolutions.

## Materials and methods

### Experimental data

Data sets that are subject to our analysis are available on-line:

Data set A: https://doi.org/10.17026/dans-zue-zz2y,
Data set B: http://www.fc.up.pt/addi/ph2%20database.html.

Because of concerns related to patient privacy and permissions set by the clinic that collected the data, some access restrictions apply to data set *A*.

Data set *A* was collected in Poland from anonymous dermoscopy patients in 2012-2013 [19]. There were 102 malignant melanoma (M) and 83 dysplastic nevus (D) cases (altogether 185 images). Most (not all) of the JPEG images were annotated with some patients data like: sex, age, and location of the lesion (this kind of information is not used in this study). All of the images referred to lesions that were resected and examined under microscope in a histopathological lab. All the histopathological examinations were performed by one experienced team of pathologists, which could prevent from contingent false positives in the sample (we investigated this problem earlier and found few misdiagnosed cases by inexperienced specialists). This is important, since the histopathological examination is the ground truth which affects assignment of the class labels.

The images were collected with resolution of 2272x1704 by Minolta Z5 digital camera with an extra dermoscopy extension and then JPEG-compressed as 3x8-bit RGB components. No magnification details were available.

On the dermoscopic images no pre-processing tasks took place. This was due to the lack of rough artifacts (black borders, hairs, droplets of immersion fluid, etc.), but also to eliminate any bias on the final wavelet base selection.

To be capable of performing the wavelet transform, the RGB representation (three integers per pixel) had to be translated into an indexed representation. This was done by linear, monotonic color mapping.

Data set *A* was used to prepare two derived data sets of the same size by (recursively) averaging neighbor pixel values in 2x2 blocks:

Images in the data set *A*2 had resolution of 1136x852.

Images in the data set *A*4 had resolution of 568x426. Since each iteration of the wavelet decomposition downscales the input image by a factor of 2 both in rows and columns, to allow for three wavelet iterations the 568x426 set was padded with zeros (rows 427 and 428).

Data set *B* was downloaded from the public database *PH*^2^ of the Automatic computer-based Diagnosis system for Dermoscopy Images (ADDI) in Portugal [20]. The *PH*^2^ database contains 200 dermoscopic images of melanocytic lesions (80 common nevi, 80 atypical nevi, and 40 melanomas) with medical annotations of expert dermatologists. The annotations refer to the dermoscopic criteria (Asymmetry: 0 = Fully Symmetry, 1 = Asymmetry in One Axis, 2-Fully Asymmetry; Pigment Network: AT = Atypical, T = Typical; Dots/Globules: A = Absent, AT = Atypical, T = Typical; Streaks: A = Absent, P = Present; Regression Areas: A = Absent, P = Present; Blue Whitish Veil: A = Absent, P = Present; Colors: 1 = White, 2 = Red, 3 = Light-Brown, 4 = Dark-Brown, 5 = Blue-Gray, 6 = Black), and clinical/histological diagnosis (0 = Common Nevus, 1 = Atypical Nevus, 2 = Melanoma). For most of the images binary masks from lesion segmentation is available.

The *PH*^2^ images were taken during follow-up examinations at the Dermatology Service of Hospital Pedro Hispano (Matosinhos, Portugal) through Tuebinger Mole Analyzer with magnification of 20x. They are 3x8-bit RGB color images with a resolution of 768x560 pixels. The image size was cropped centrally to 760x552 pixels for the sake of the aforementioned wavelet recursive transformation.

Since data set *A* contained only melanoma and displastic (atypical) lesions, we limited data set *B* to similar (not identical) medical cases. Finally data set *B* had the following structure: 40 melanomas (33 from clinial + histopathological, 7 lesions from only clinical examination), 77 common/dysplastic nevi (5 from clinial + histopathological, 72 lesions from only clinical examination). Altogether in *B* 113 lesion images were analyzed.

In order to directly compare the efficiency of the SVM classifiers between data set A and B, the latter was additionally resized to resolutions A/A2/A4 and a series of experiments with the mixed data set A + B was done.

Clinical statistics show that the incidence rate of melanoma is up to 5% (on average) within all ‘suspicious’ pigment lesions [21]. This means that the melanoma class is the minority class and is under-represented compared to the benign class. This status holds in the clinic, whereas in the melanoma CAD scientists attempt either to balance the number of cases from both classes (data set *A* is about this status) or to introduce sub- or over-sampling techniques [22] (this is required in *B*).

In this work we took for both the data sets *A* and *B* the whole statistics for the minority class (melanoma) and experimented with different sub-sampling ratios [23] of the benign lesions. As in [8] for the data set *A* no major change in the classification performance (<5% for *B*) was observed. This estimation allows us to conclude that the data imbalance problem in our experiment does not impact noticeably. In fact, in *B* the class ratio is 1:2, which is not a dramatic imbalance.

Disregarding the way how imbalance is removed/controlled in this or (generally) other ML experiments, the problem in the clinic still holds, because a question arises how majority cases are picked out for the data set. It follows from our experience that usually those images are selected which visually fits in very well with the set of benign (dysplatic) cases. This may, however, seriously impact ‘production’ classifiers. We conclude that bias comes preferably from the ‘clinical’ source and not our data statistics or procedures.

### Wavelet features

Skin is texture that manifests repetitive patterns, pigment network and different structures (globules, streaks, etc.). This reality can be analyzed in a range of frequencies and spatial scales, which goes beyond pure Fourier analysis. In this section we introduce wavelet features that are used in this work.

The Discrete Wavelet Transform (DWT) decomposes a signal into a coarse (average) signal and a signal of details. This can be interpreted, after Mallat [24], in terms of lowpass (L) and highpass filters (H). The subsequent levels of DWT operate recursively on the lowpass (scaling) part of the output. Products of the transform are downsampled (decimated) by two at each level.

In the wavelet packet transform (WPT), the filtering operations are also applied to the signal of details. This results in a substantial amount of subbands in the output and makes the time (space) vs. frequency analysis much finer than in DWT. Multiresolution analysis with WPT can reveal scale-based features of the signal, which provides more precise information than other signal analysis techniques. Our working hypothesis is that WPT suits well to determine distinctive features characterizing the class of the dermoscopy image.

Images are two-dimensional signals so one iteration of the Mallat filtering algorithm produces four sub-signals which can be considered as LL, LH, HL and HH filters after 1D wavelet transform on the rows and then on the columns. Those filters can be interpreted as decimated sub-images.

We used three iterations of WPT.

**Fig 1:**
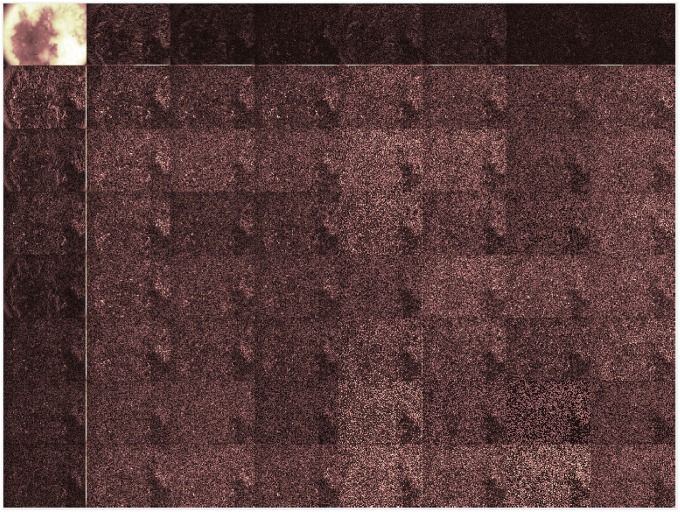
The wavelet packet transform (WPT) decomposition.

Since all wavelet filters are downsampled (by a factor of 2) after each run of the wavelet transform (in rows and columns separately), four subimages were produced. In Fig 1 a summary image with all the used stages of decomposition is shown. It is evident that all channels of decomposition are used (WPT).

In one iteration four filters were produced, so in three turns we had 1 + 4 + 16 = 21 different transformation branches. In each branch we produced a list of twelve features. As features we took simple measures based on the energy content of the (sub)images [11, 12]:

*e*_*i*_—energies,
*e*_*i*_/*e*_*max*_—maximum energy ratios,
*e*_*i*_/Σ*e*_*k*_, *k* ≠ *i*—fractional energy ratios,

where energy *e*_*i*_, *i* = 1, 2, 3, 4 was defined as a sum of absolute values of the pixels. Our feature vector was composed of 21 × 12 = 252 components.

Such feature extraction was carried out in different wavelet bases: orthogonal wavelets: Haar, Daubechies, Symlets, Coiflets, bi-orthogonal wavelets, and reverse bi-orthogonal wavelets. Those wavelet bases possess certain characteristics which however is not discussed in this article.

### SVM learning

The Support Vector Machine (SVM) learning method finds a hyperplane that separates data points from two distinct classes in the way that it maximizes the margin between the two classes [25, 26]. The mathematical formulation of the problem [27] involves finding *β* and *b* such that for all the data points (*x*_*i*_, *y*_*i*_) it holds:

$$\begin{array}{c} f\left(x\right)=x^{\prime }\beta +b=0,\;y_{i}f\left(x_{i}\right)\geq 1 \end{array}$$ (1)

where the latter equality is accomplished for those data points that are (called) support vectors (*y*_*i*_ *f*(*x*_*i*_) = 1). In a case the two classes are not separable by a hyperplane SVM introduces a soft margin i.e. it releases the condition *y*_*i*_ *f*(*x*_*i*_) ≥ 1 − *ξ*_*i*_. The optimization problem formulated by Lagrangian (in the simplest form of the *L*^1^ norm):

$$\begin{array}{c} L=\frac{1}{2}\beta^{\prime }\beta +C\sum_{i}\xi_{i}-\sum_{i}\alpha_{i}\left(y_{i}\left(x_{i}^{\prime }\beta +b\right)-\left(1-\xi_{i}\right)\right)-\sum_{i}\mu_{i}\xi_{i} \end{array}$$ (2)

with the bounds:

$$\begin{array}{c} \beta =\sum_{i}\alpha_{i}y_{i}x_{i},\;\sum_{i}\alpha_{i}y_{i}=0,\;\alpha_{i}=C-\mu_{i},\;\alpha_{i},\mu_{i},\xi_{i}\geq 0 \end{array}$$ (3)

is usually translated into an expression with the *α* coefficients only:

$$\begin{array}{c} max_{\alpha }\sum_{i}\alpha_{i}-\frac{1}{2}\sum_{i}\sum_{j}\alpha_{i}\alpha_{j}y_{i}y_{j}x_{i}^{\prime }x_{j} \end{array}$$ (4)

with ∑_*i*_ *α*_*i*_ *y*_*i*_ = 0 and 0 ≤ *α*_*i*_ ≤ *C*, C being the regularization parameter.

This concept is very successful in machine learning and data mining applications [28], especially when non-linear transformations of the primary data space (kernels) are introduced.

The appropriate choice of kernel can map primary features to a larger dimensional space where the classes are (better) separable. Four kernels are well known and widely used: the linear kernel $x_{i}^{T}x_{j}$, which is a linear combination of features, the polynomial kernel ${\left(x_{i}^{T}x_{j}+r\right)}^{p}$ with the parameters: p-degree of polynomial and r-offset, the gaussian (radial basis) kernel *exp*(−*γ* * |*x*_*i*_ − *x*_*j*_|^2^), *γ* > 0, and sigmoid (MLP) kernel.

Quadratic programming which is required to solve the optimization problem defined above is broadly implemented in various statistical toolboxes and professional computer libraries e.g. in [29]. In this study we take advantage of both the LIBSVM library [29] and Matlab Statistical Toolbox [30]. We take into account the binary SVM classifiers with the linear, polynomial, and gaussian kernel.

The SVM paradigm, as compared to standard back-propagated neural networks, does not have hidden parameters, and is a global approach not prone to stuck at local minima. Different bounds set on the margin can affect the (upper bound of) generalization error and can control overfitting.

### Bayesian optimization

The objective of every learning method is to minimize a cost function, which is usually the cross-validated loss. In the learning paradigm exploited in this work, i.e. C-SVM, we want to search for the best parameters of the kernel and the domain size C, as well as the number of support vectors in the resulting model. One framework to minimize a real-valued objective function within a certain box is Bayesian Optimization (BO) [31].

BO is a global optimization technique (starting points are of no importance) but there are cases where it does not always secure accurate results. If no a priori knowledge about parameters is available, the widest finite range should be covered. BO algorithm is time-consuming due to large computational burden every iteration, therefore for the sake of experimental requirements it should stop if the cross-validated error rate (objective function) drops below a certain level or (at least) after reaching a fixed number of iterations, or maximum run time.

BO introduces the so called acquisition function, that determines which points of the space should be covered. ‘Goodness’ of the point is evaluated upon the ‘amount’ of improvement it provides for the optimization. There can be different methods to achieve this and our model of choice is based on the posterior distribution function *Q*.

If at *x*_*best*_ the lowest value of the posterior mean is *μ*_*Q*_(*x*_*best*_) then the ‘expected improvement’ is:

$$\begin{array}{c} EI\left(x,Q\right)=E_{Q}\left[max\left(0,\mu_{Q}\left(x_{best}\right)-f\left(x\right)\right)\right] \end{array}$$ (5)

To escape from local minima of the objective function, in order to balance sampling at points with high and low efficiency of the model, the acquisition function checks after each iteration the standard deviation of the posterior distribution function $\sigma_{Q}^{2}\left(x\right)$. If it drops below a defined level, the area is recognized as ‘overexploited’ and the algorithm modifies the kernel to raise the variance *σ*_*Q*_. This offsets the solution to a new not yet examined point. Such strategy both covers the whole space and concentrates on the best feasible points.

## Results and discussion

Our setup was coded first with LIBSVM [29] and then recoded to Matlab [30]. The experiments ran on a 4-core i7 workstation with 32GB RAM. We cross-validated (10-fold CV) our SVM classifiers on the standardized (*N*(0, 1)) predictor vectors.

Search for the best subset of features is usually a tradeoff between reducing the bias and computational cost, and preserving the classification performance. In this work we did not reduce our feature vector and kept all the wavelet features in the analysis. Our objective was to finally compare different learning methods, so we had to remove any bias coming from feature selection methods, which can affect the analyzed learning paradigms in a different way. This was discussed in [32] where feature selection algorithms (CFS, PCA, GSFS) reduced the complexity of the classification, but performance was highly dependent upon the classifier.

**Fig 2:**
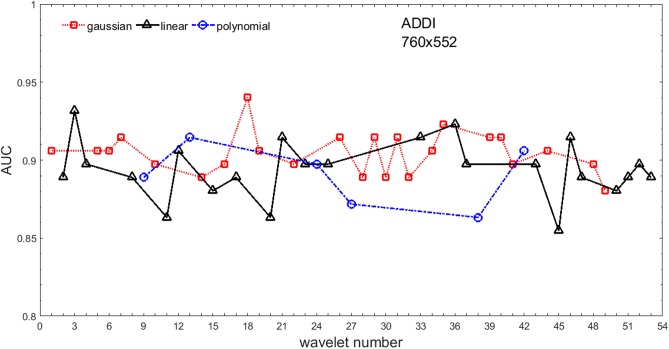
AUC as a function of the wavelet number (see text) for data set *A* for the three SVM kernels optimized by the Bayesian search. Resolution is (2272x1704). To easily follow the trend the points belonging to the same kernel function are connected with a line.

**Fig 3:**
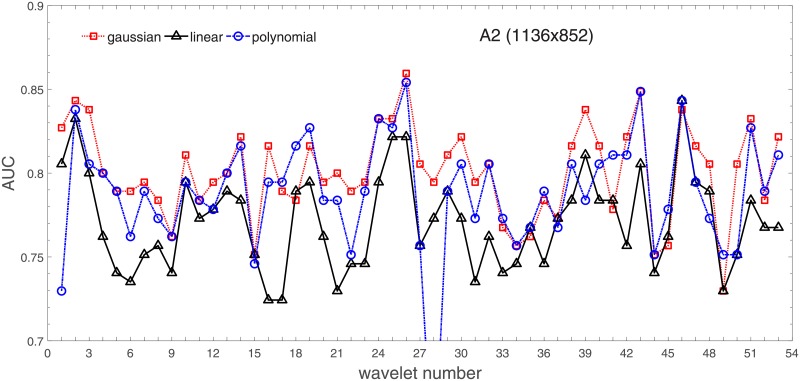
AUC as a function of the wavelet number for data set *A* for the three SVM kernels optimized by the Bayesian search. Resolution is A2(1136x852).

**Fig 4:**
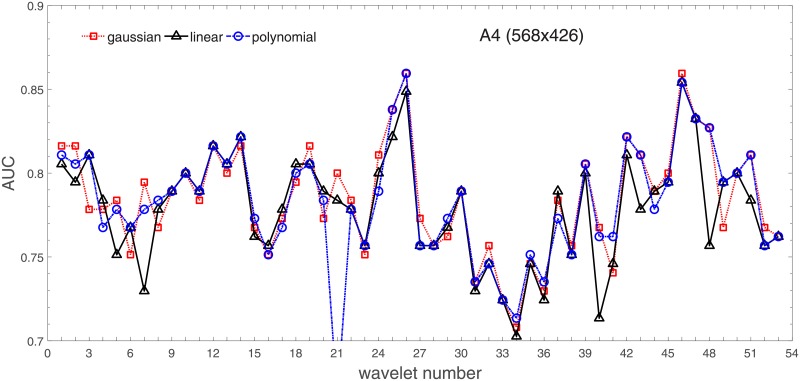
AUC as a function of the wavelet number for data set *A* for the three SVM kernels optimized by the Bayesian search. Resolution is A4(568x426).

**Fig 5:**
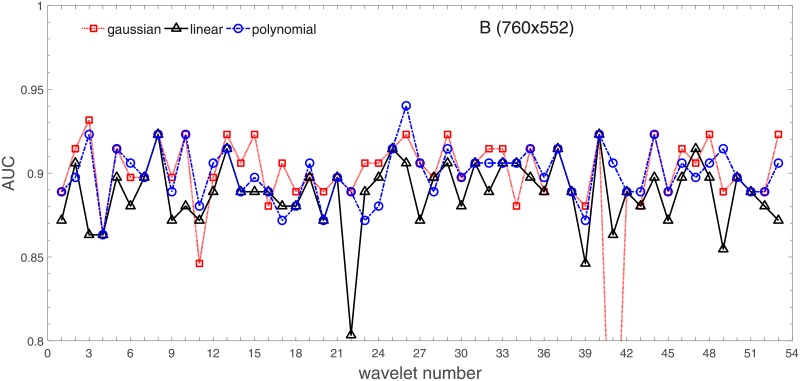
AUC as a function of the wavelet number for data set *B* (original resolution 760x552) for the three SVM kernels optimized by the Bayesian search.

**Fig 6:**
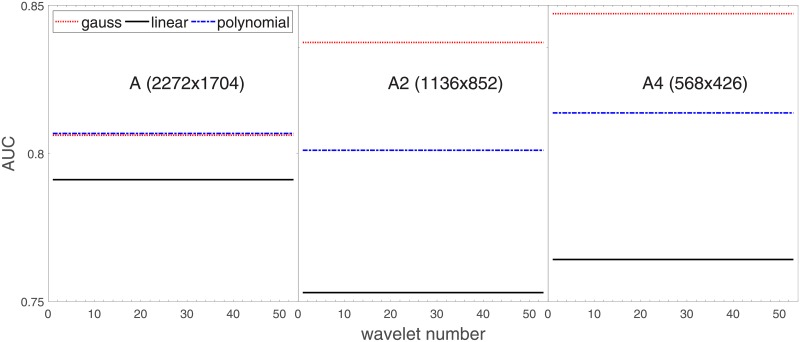
Average AUC levels for the three kernel functions, for data set *A* (resolutions *A*, *A*2, *A*4).

**Fig 7:**
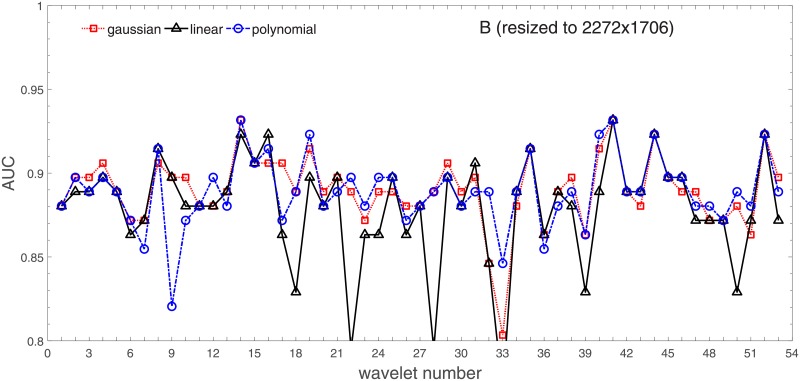
AUC as a function of the wavelet number for data set *B* for the three SVM kernels optimized by the Bayesian search. Resolution is resized to (2272x1704).

**Fig 8:**
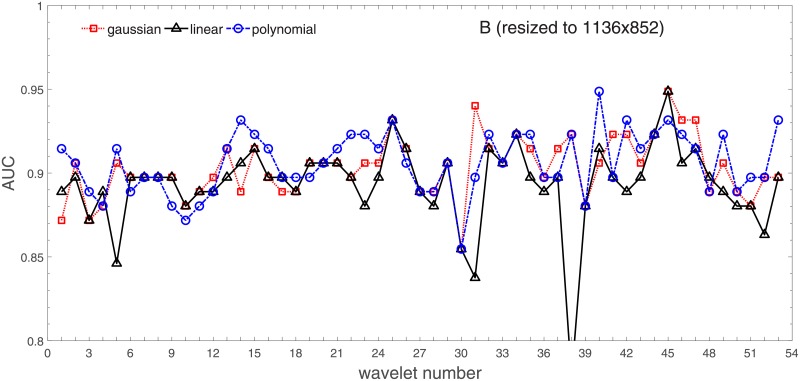
AUC as a function of the wavelet number for data set *B* for the three SVM kernels optimized by the Bayesian search. Resolution is resized to (1136x852).

**Fig 9:**
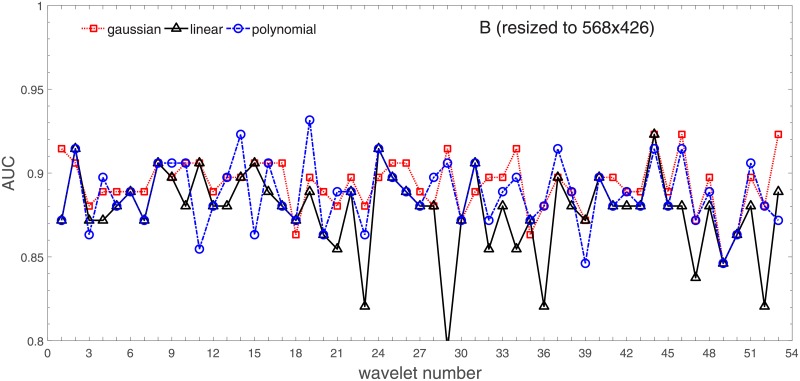
AUC as a function of the wavelet number for data set *B* for the three SVM kernels optimized by the Bayesian search. Resolution is resized to (568x426).

**Fig 10:**
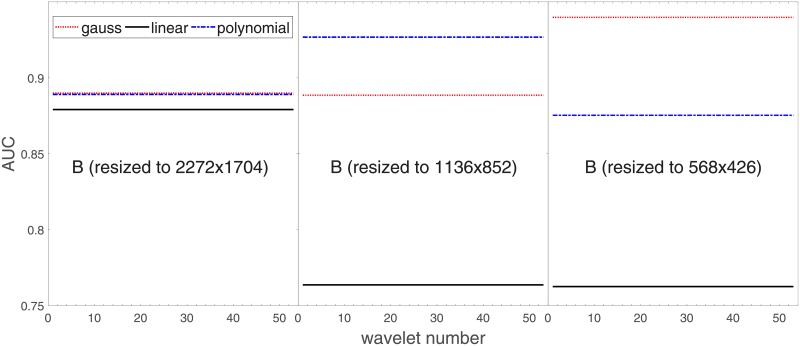
**Average AUC levels for the three kernel functions, for data set *B* resized to resolutions:** *A*, *A*2, *A*4.

**Fig 11:**
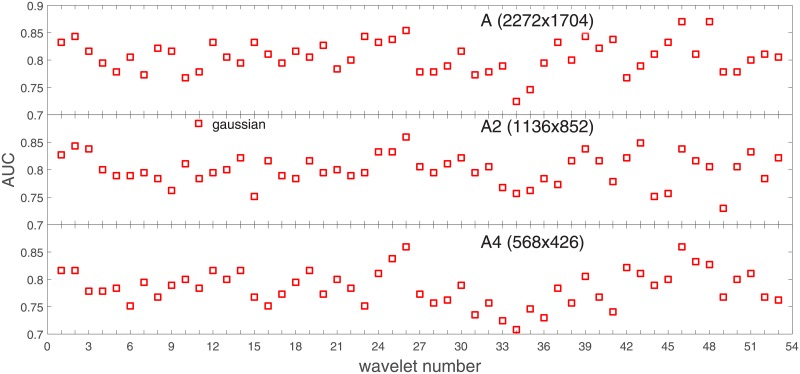
AUC for the SVM gaussian kernel as a function of the wavelet number for the data set *A* (resolutions: *A*, *A*2, *A*4).

**Fig 12:**
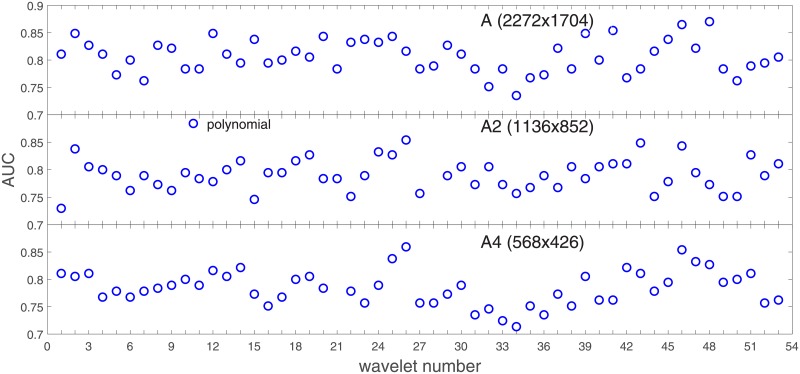
AUC for the SVM polynomial kernel as a function of the wavelet number for the data set *A* (resolutions: *A*, *A*2, *A*4).

**Fig 13:**
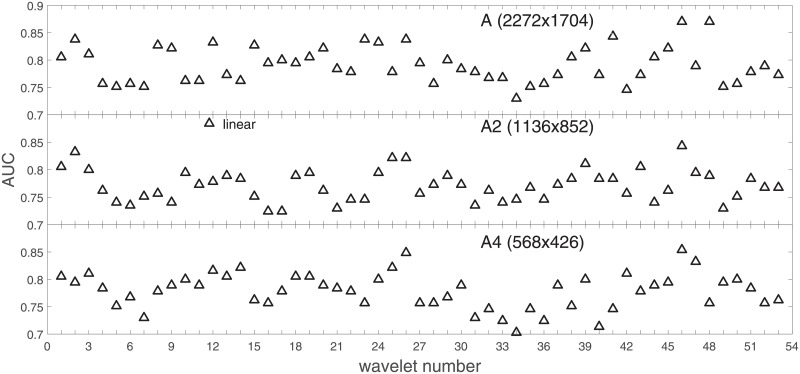
AUC for the SVM linear kernel as a function of the wavelet number for the data set *A* (resolutions: *A*, *A*2, *A*4).

**Fig 14:**
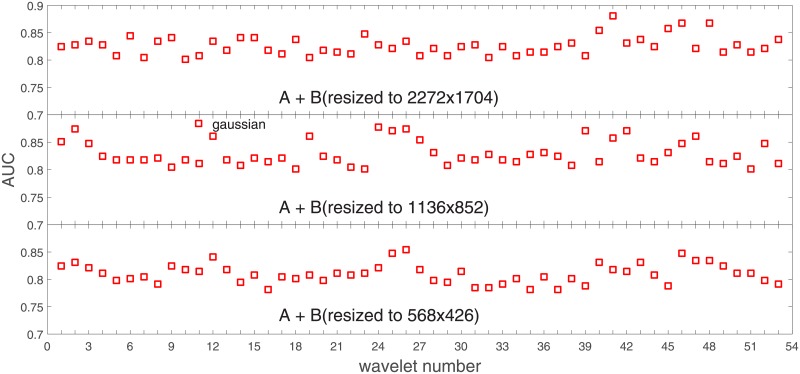
AUC for the SVM gaussian kernel as a function of the wavelet number for the combined data set *A* + *B* (*B* is resized to *A*, *A*2, *A*4).

**Fig 15:**
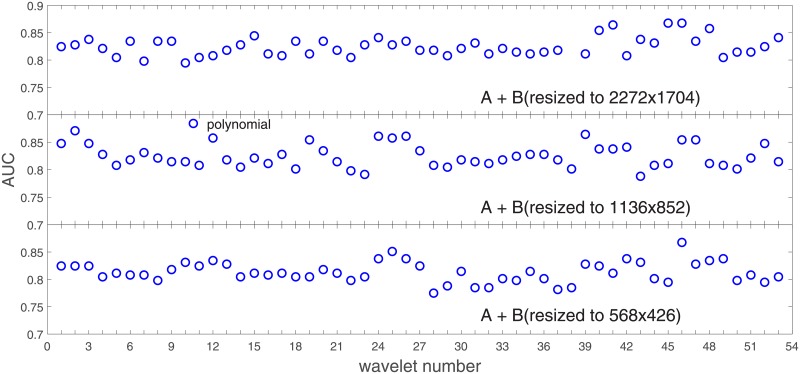
AUC for the SVM polynomial kernel as a function of the wavelet number for the combined data set *A* + *B* (*B* is resized to *A*, *A*2, *A*4).

**Fig 16:**
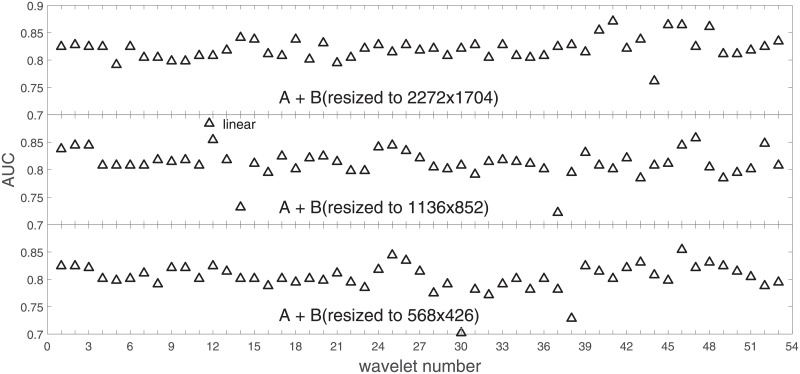
AUC for the SVM linear kernel as a function of the wavelet number for the data set *A* + *B* (*B* is resized to *A*, *A*2, *A*4).

**Fig 17:**
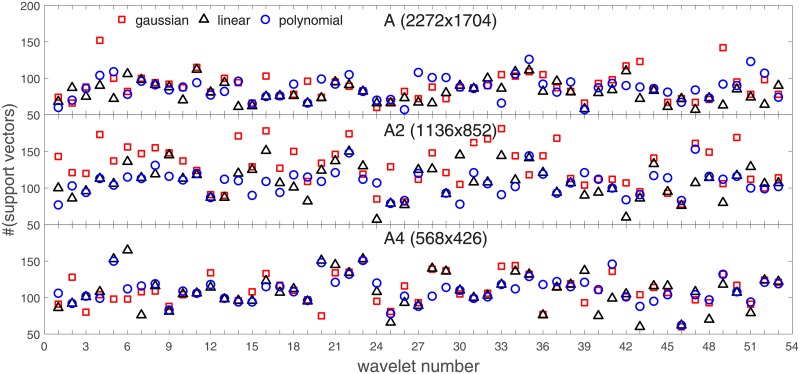
Number of support vectors for the three kernel functions used in the SVM classifier of data set *A*.

**Fig 18:**
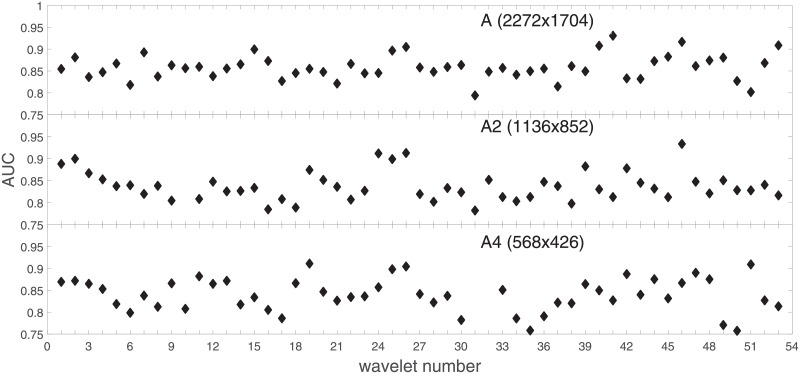
AUC for the ensemble learning optimized by the ‘recall’ measure as a function of the wavelet number for data set *A* (resolutions: *A*, *A*2, *A*4).

**Fig 19:**
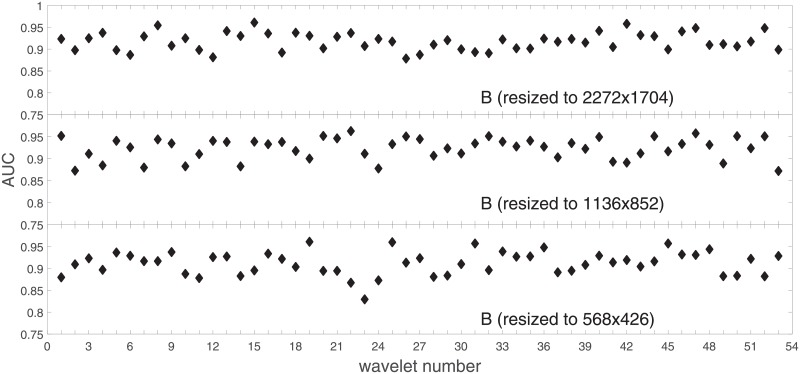
AUC for the ensembling learning as a function of the wavelet number for data set *B* resized to resolutions *A*, *A*2, *A*4.

**Fig 20:**
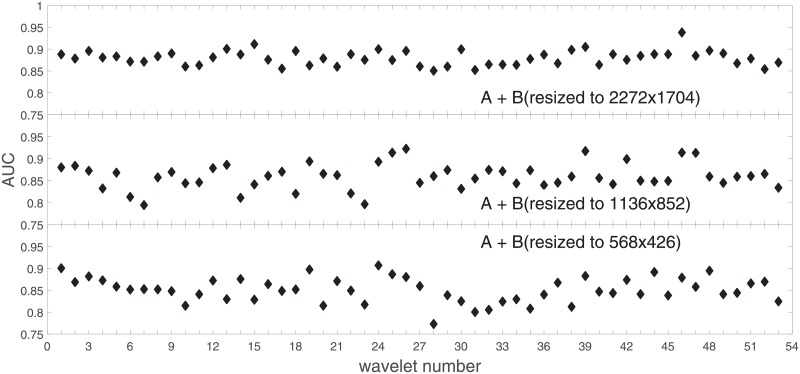
AUC for the ensembling learning optimized by the ‘recall’ measure as a function of the wavelet number for data set *A* + *B* (*B* is resized to *A*, *A*2, *A*4).

**Fig 21:**
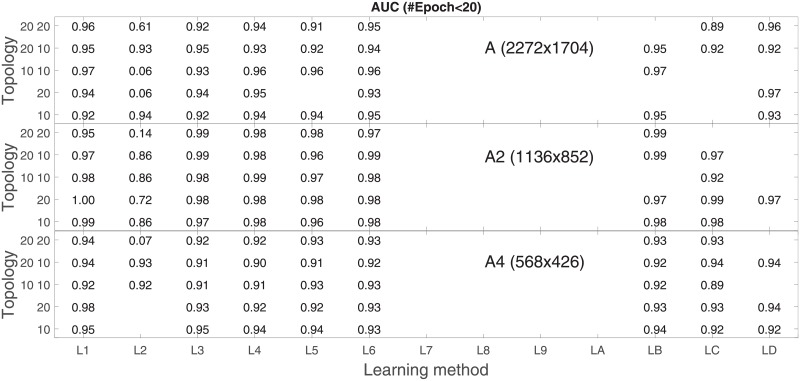
AUC for the neural feed-forward classifier as a function of the topology and learning function (see text for the details). Data set is *A* in the native *A* and reduced resolutions *A*2 and *A*4.

**Fig 22:**
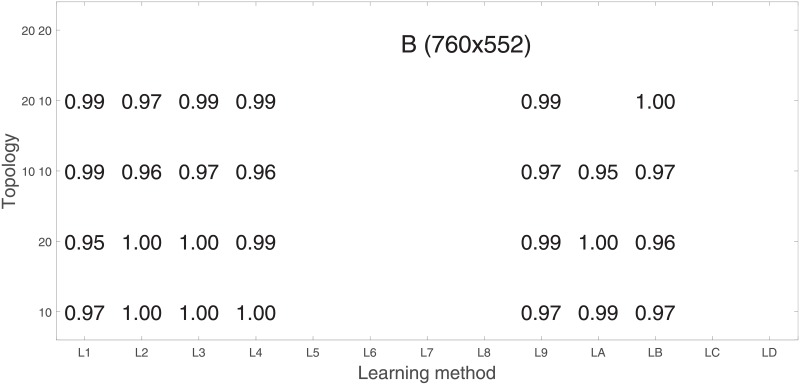
AUC for the neural feed-forward classifier as a function of the topology and learning function (see text for the details). Data base is *B* in the native resolution 760x552.

**Fig 23:**
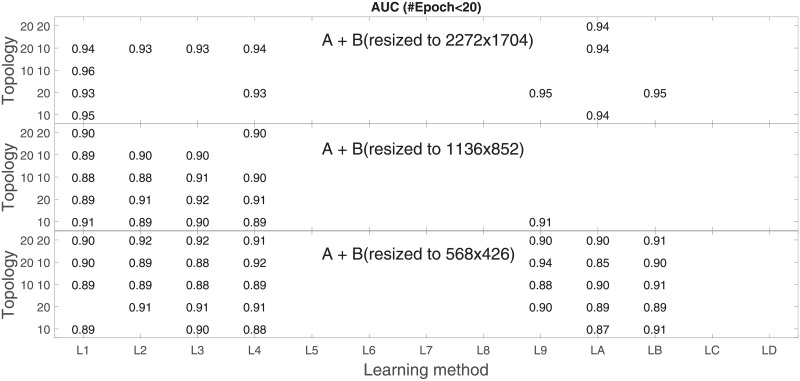
AUC for the neural feed-forward classifier as a function of the topology and learning function. Data base is *A* + *B* (*B* is resized to the native and reduced resolutions of the data base *A*).

Analysis and discussion of our results is divided into several stages:

1. Analysis of the absolute SVM classification performance for all the kernel functions for data set *A* (*A*/*A*2/*A*4) and data set *B* (original resolution), which includes Figs 2–5. Our objective is to analyze an overall classification performance of the kernel functions. In Fig 6 we analyze average AUC levels for the gaussian, linear, and polynomial kernels.
  We also present classification performance for all the kernel functions for data set *B* resized to resolutions *A*/*A*2/*A*4 (Figs 7–9). Again, in Fig 10 we analyze average AUC levels for the three kernels.
2. Analysis of the classification performance for a fixed kernel function for three image resolutions for data set *A* (Figs 11–13) plus for the combined data set *A* + *B*(*resized*) (Figs 14–16). Our objective here is to pick up those wavelet bases that show outstanding classification performance for individual data sets with specific image resolutions. Our second objective is to select those wavelet bases that preserve high classification efficiency through the presented spectrum of resolutions (resolution invariance).
  Analysis of the joint data set *A* + *B* cast light both on compatibility of the data sets and stability of the learning algorithm (independence on the data set).
  In Fig 17 we show the number of support vectors for the three kernel functions used in the SVM classifier of data set A. This is also a test for quality and stability of features built upon certain wavelet numbers.
3. Analysis of our SVM results against our previous experiments with the wavelet features: ensembling (Figs 18–20) and feed-forward neural networks classifiers (Figs 21–23). Our objective is to show the property of the resolution invariant wavelet features for melanoma detection in the background of other tested classification paradigms.
4. Analysis of our SVM results with help of the melanoma CAD summary articles (Masood [3], Oliveira [4]) and some individual studies from the literature.

### Ad.1

Full results of our experiments on data set *A*/*A*2/*A*4 are presented in Figs 2–4 for the three kernel functions and the three resolutions. In these and the follow-up figures the wavelet number denotes: 1 = Haar, 2-11 = Daubechies (db1-db10), 12-18 = symlets (sym2-sym8), 19-23 = coiflets (coif1-coif5), 24-38 = bi-orthogonal wavelets (Bior1.1, 1.3, 1.5, 2.2, 2.4, 2.6, 2.8, 3.1, 3.3, 3.5, 3.7, 3.9, 4.4, 5.5, 6.8), and 39-53 = reverse bi-orthogonal wavelets (Rbio1.1, 1.3, 1.5, 2.2, 2.4, 2.6, 2.8, 3.1, 3.3, 3.5, 3.7, 3.9, 4.4, 5.5, 6.8).

The way how images, so 2D signals, are analized by the filter bank is another factor. In this work 1Dx1D filtering (separately along rows and columns) was performed, which followed Mallat [24] (in other approaches 2D filtering elements are potentially possible).

For the SVM optimization we used the Sequential Minimal Optimizer [31] and initiated the alpha coefficients with zeros. To check for the optimization convergence the gradient tolerance was set for 10^−3^. The AUC values were averaged over 10-fold cross-validation (10 turns, 9 training subsets and 1 hold-out testing subset in each turn).

The optimizatoin space that was checked included:

- kernel functions: gaussian, polynomial, linear,
- parameters of the kernel functions: for ‘gaussian’ it is the inverse variance parameter (*γ*), for ‘polynomial’ it is the order of the polynomial (d),
- the maximum penalty imposed on margin-violating data points (*C*).

We scanned the above-mentioned model parameters in a log step between 1e-3 and 1e6. The maximum number of iterations was fixed for 60 and the run time was left infinite.

Figs 2–4 show AUC (the Area Under the ROC Curve) vs. the wavelet number for data sets *A*(2272x1704), *A*2(1136x852), and *A*4(568x426) for the binary SVM classifiers of melanoma vs. displastic lesion. This is a Bayesian search over an available space of the gaussian (marker is square), linear (triangle), and polynomial (circle) kernel.

Clearly visible is an inphase sinusoidal fluctuation of AUC through all the resolutions. Since all the kernel functions fluctuate over the wavelet number in a similar way (the trend, not the absolute level) this must be the feature set that is responsible for the phenomenon, not the learning paradigm. Indeed, variability in the classification performance comes both from the individual wavelet base, and is also present within each wavelet family. The latter is obvious since wavelets from one wavelet family differ in scale and offset. The skin texture apparently ‘prefers’ some scales.

The wavelets that were used in the analysis have different properties like smoothness, symmetry, or support. The wavelet base complies with some model constraints and has a diverse impact on the texture classification. Haar, Daubechies, symlets, and coiflets are orthogonal wavelets. Orthogonal wavelets are energy preserving and non-redundant, which follows the formula *X*^2^ = *A*^2^ + *D*^2^ (*X*-image, *A*-reconstructed image of approximation, and *D*-reconstructed image of details). After Mallat [24] wavelets can be represented by a filter bank. In this case this is a pair of a single scaling function and a wavelet.

The bi-orthogonal wavelet filters, on the other hand, generate one scaling function and a wavelet for decomposition, and another pair for reconstruction. They have the property of perfect reconstruction i.e. *X* = *A* + *D* and a linear phase, which makes them more symmetric and compact (these properties are useful in signal analysis). For the sake of symmetry we can divide the selected wavelet bases into: symmetric functions (Haar and (reverse)-biorthogonal wavelets), nearly symmetric functions (symlets, coiflets), and asymetric functions (Daubechies). Taking into account this variety of properties we conclude that they are individual wavelets, not wavelet families that make the SVM classification of melanoma dermoscopy images more (or less) efficient, although certain wavelet families have a higher number of ‘efficient’ wavelets than the others.

Performance of the SVM classification on data set *B* for the three kernels is shown in Fig 5. Images from this data set have rather low resolution but (unexpectedly) they yield (average) AUC values about 10% higher. Two factors may play a role:

Compression: Data set A is a collection of JPEG images compressed by a lossy algorithm. Rough estimation on the information content of an image from data set A is possible from its JPEG file size (assumed we skip all the headers from different stages of the compression). We have the following (average) file sizes: 1.35MB (*A*, 2272x1704), 0.59MB (*A*2, 1136x852), and 0.17MB (*A*4, 568x426). This yields: 2.79 bit/pix, 4.89 bit/pix, and 5.45 bit/pix from the highest to the poorest resolution, respectively.

Data set B, in turn, has (standardized) resolution of 760x552 and the average file size is 1.33MB. This yields information capacity of about 24.26 bit/pix. Such an extremely high information content is due to its lossless file format, which is BMP. Even if we compare data set *B* only to the lowest resolution data set *A*4, the factor between them is about five. We know that the JPEG algorithm has a quantization stage which ‘flattens’ pixels values based on the JPEG quality/compression ratio. This may affect the image structure which translates to decreased sensitivity for patterns in the compressed image. Quantitative estimations between data sets *A* and *B* are not possible as far as (pre)processing steps in *B* are unknown.

Class content: The existing melanoma pattern recognition methods (a brief comparison is done later in this article) are rather ‘advanced’ systems so are ‘costly’ in terms of feature extraction/selection and classification. This reflects the ‘clinical’ difficulty in discriminating melanoma from displastic (anomal) skin lesions. Data set *A* has two distinct classes: melanoma and dysplastic nevus while data set *B*, in turn, has class melanoma and a mixture of dysplastic (1/3) and common (2/3) nevus. While the benign class in *B* (common + dysplastic nevus) must be more widely distributed in the feature space than dysplastic cases alone (*A*), the ‘distance’ between the melanoma and this (mixed) class in *B* should be larger i.e. more efficient for classification, than in *A*.

Those two factors may be, though qualitative, but reasonable explanation for the difference in the average AUC level between the data sets *A* and *B*.

Since there are a few variables in our SVM experiments (the wavelet bases, different resolutions, and finally the three kernel functions) in order to interpret the results and draw straight-forward conclusions we will limit the number of factors and answer the question which kernel function produces the best average classification performance in terms of AUC.

Fig 6 has three sections in which we show an average level of AUC produced by the SVM classifiers with the appropriate kernel functions: gaussian (dotted line), polynomial (dash-dotted line), and linear (solid line) for the nominal *A* and two downgraded resolutions *A*2 and *A*4. The polynomial kernel function keeps its average level in the reduced image resolutions while the liner one looses classification efficiency considerably. An interesting finding is that the gaussian kernel rises continuedly towards lower resolutions. Beyond this point (568x426), when we go further with the resolution reduction the classification performance drops rapidly to a level of about 70%.

For the polynomial and linear kernel we can see a drop in performance for the intermediate resolution (1136x852) and then they recover loss and even outrun a little bit in 568x426. This behavior is independent of the absolute AUC level, this happens also for the linear kernel whose performance beyond the nominal 2272x1704 is poor.

In order to check whether those average levels are data set specific (*A*) or general, we resized images from the data set *B* to the resolutions of the data set *A*. This can be found in Figs 7–9.

Based on the numerical results of Figs 7–9 we could depict in Fig 10 an average level of AUC produced by the SVM classifiers with the same kernel functions (gaussian, polynomial, and linear) for data set *B* resized to resultions *A*, *A*2, and *A*4. Again, the linear kernel has a decreased (average) level of AUC along with the reduced resolution. As in *A* the gaussian kernel performs better for (568x426), but slightly worse than the polynomial kernel for (1136x852). Taking into account statistical aspects of learning and possible performance drops for (1136x852) apparent in *A*, both Figs 6 and 10 look similar.

From this analysis we can conclude the following:

1. kernel functions play a key role in the performance of the SVM classification. The poorest linear kernel (dot product of the feature vector components) cannot appropriately map features-related (noisy) classes of melanoma and benign cases. The polynomial kernel, which can translate the primary feature vector into more dimensional space (degree of the polynomial is a parameter of the model) performs better, but the gaussian kernel outperforms all the other. This is because it provides the feature vector mapping in a more nonlinear fashion through introducing cluster-like subspaces. Apparently this is suitable for the SVM classifiers for the melanoma detection.
2. wavelet features classified by SVM with different kernel functions have ‘preferential’ resolutions where they reach the most optimal classification efficiency. Our analysis on two data sets (*A*, *B*) show that for the gaussian kernel the most efficient space for the SVM classifier is this of 568x426. This observation is important since can affect the computational aspects of the melanoma classification. Low(er)-resolution image analysis would be potentially resource-saving without (noticeable) degradation of the classification performance.

### Ad.2

In stage 1 we performed an analysis of the average SVM classification performance for all the kernel functions for two data sets: *A* and *B*. We concluded that tha gaussian kernel outperforms the polynomial and linear kernel especially towards lower image resolutions and that both data sets yield compatible results.

In stage 2 we focus on the gaussian kernel function and analyze the absolute classification performance (in terms of AUC) for the three image resolutions (2272x1704, 1136x852, 568x426) in *A* and the combined data set *A* + *B*.

Our first objective here is to pick up the wavelet bases that show outstanding classification performance for the individual data sets with specific image resolutions. The second objective is to select those wavelet bases that preserve high classification efficiency through the presented spectrum of resolutions (resolution invariance).

Analysis of the joint data set *A* + *B* should prove both compatibility of the data sets and stability of the learning algorithm (independence on the data set). Although our analysis is focused on the gaussian kernel (*A*: Fig 11, *A* + *B*: Fig 14), we present results for the other two kernel functions as well (*A*: Figs 12 and 13, *A* + *B*: Figs 15 and 16). This should cast light on robustness and stability of the wavelet features.

Below we present for the individual kernel functions some consolidated figures with the three image resolutions. In each resolution we select a set (we arbitrarily choose n = 6) of wavelet bases that show an outstanding AUC classification performance. In Table 1 we call it ‘Condition 1’. In ‘Condition 2’ we require that the wavelet number has the highest AUC.

**Table 1:** Wavelet numbers collected for the kernel functions (gaussian, polynomial, linear) satisfying some conditions for the AUC values (numbers in parentheses have equal AUC values). Condition 1: outstanding SVM classification efficiency. Condition 2: highest classification efficiency. Condition 3: high classification efficiency in all the resolutions (order of priority in A). Condition 4: high classification efficiency in all the resolutions (order of priority in A2). Condition 5: high classification efficiency in all the resolutions (order of priority in A4).

Data Set	Resolution	Condition 1	Condition 2	Condition 3	Condition 4	Condition 5
gaussian						
A	2272x1704	(**46** 48) *26* (39 2 23)	**46**	**46** 48 *26* 2 39 12		
A2	1136x852	*26* 43 (2 39 **46** 3)	*26*		*26* 2 **46** 39 19 30	
A4	568x426	(**46** *26*) 25 47 48 42	**46**			**46** *26* 48 2 19 12
B	760x552	3 (8 10 13 15 *26*)	3	-	-	-
A + B	2272x1704	41 (**46** 48) 45 40 23	41	41 **46** 48 40 6 9		
A2 + B2	1136x852	24 (*26* 2) 39 25 42	24		24 2 *26* 25 12 47	
A4 + B4	568x426	*26* (**46** 25) 12 (47 48)	*26*			*26* **46** 25 12 48 2
polynomial						
A	2272x1704	48 **46** 41 39 12 2	48	**46** 39 2 25 *26* 19		
A2	1136x852	*26* 43 **46** 2 24 19	*26*		*26* **46** 2 25 19 39	
A4	568x426	*26* **46** 25 47 48 42	*26*			*26* **46** 2 19 39 25
B	760x552	*26* (40 44 10 3 8)	*26*	-	-	-
A + B	2272x1704	**46** 45 41 48 53 15	**46**	**46** 24 9 *26* 20 25		
A2 + B2	1136x852	2 39 *26* 24 12 25	2		24 *26* 25 19 2 9	
A4 + B4	568x426	**46** 25 42 49 *26* 24	**46**			**46** 25 24 *26* 2 9
linear						
A	2272x1704	**46** 48 41 23 *26* 2	**46**	**46** *26* 12 2 39 19		
A2	1136x852	**46** 2 *26* 25 39 1	**46**		**46** 2 *26* 39 19 18	
A4	568x426	**46** *26* 47 25 14 12	**46**			**46** *26* 19 18 12 39
B	760x552	(40 8) (25 37 47 13)	40	-	-	-
A + B	2272x1704	41 **46** 45 48 40 14	41	**46** 48 41 *26* 15 25		
A2 + B2	1136x852	47 12 52 **46** 25 2	47		**46** 24 *26* 3 2 39	
A4 + B4	568x426	**46** 25 *26* 43 48 1	**46**			**46** *26* 2 3 48 43

As a next point we analyze high-AUC wavelet numbers almost constant in all the three resolutions. Our ‘algorithm’ to choose such cases is:

step 1: for *i* ∈ {*wavelet numbers*} calculate *SUM*(*i*) = ∑_*j*=1,2,3_ *AUC*_*i*_(*resolution*_*j*_)
step 2: choose n (n = 6) consecutive numbers: $i={\text{argmax}}_{i}SUM_{i}$

Conditions 3,4,5 in Table 1 select wavelet numbers that show high efficiency in all the resolutions (assumed a data set and a kernel function). In such a set the order of priority of the wavelet numbers depends on the resolution preference of the analyzed dermoscopy images. In ‘Condition 3’ preferential is resolution 2272x1704, in ‘Condition 4’ 1136x852, and in ‘Condition 5’ 568x426.

Table 1 shows the following:

1. Two most frequent wavelet numbers are marked: 46-in bold face, 26-in italic.
2. Wavelet numbers producing high AUC (‘Condition 1), the best one included (‘Condition 2’), belong for the most part to the Bi-orthogonal (24-38) and Reverse Bi-orthogonal (39-53) wavelet families,
3. wavelet numbers exhibiting good resolution invariance (keeping high AUC results in the three resolutions) come out in all the kernel functions (‘Condition 3,4,5’).

Some auxiliar information about how different wavelets ‘fit’ to the classifier can also be found in Fig 17. ‘Good’ wavelets from Table 1 (from the classification performance point of view) have a (preferably) small number of the support vectors. They are also less dispersed both between different kernel functions and different image resolutions. The perfect case *waveletnumber* = 46 shows very small values of the support vectors and almost perfect independence of the kernel function. This can be attributed to good quality and stability of features built upon wavelet number 46.

Collective behavior of all the wavelet bases measured both in AUC and in the number of support vectors (Fig 17) casts light on how the SVM classifier ‘prefers’ the studied resolutions. It seems that the nominal resolution 2272x1704 and 568x426 are comparable in terms of the classification performance and stability, but 1136x852 is apparently worse (in Fig 17 see the dispersion in the number of support vectors between the kernel functions).

After analysis of Table 1 we come to some conclusions regarding the SVM classification of the dermoscopy images (class melanoma vs. class displastic/common nevus) with the presented wavelet features.

First, we can see that efficient SVM learning of dermoscopy images is possible with some selected wavelet bases. Most of the high-performance wavelet bases belong to the symmetrical, bi-orthogonal or reverse bi-orthogonal wavelet families.

Second, there are some wavelet numbers which keep the SVM classification performance on an almost stable level through a range of resolutions. The two wavelet bases that show best performance in all the studied resolutions are: 46 (RBio3.1), and 26 (Bior1.5).

### Ad.3

In this section we compare our SVM results for data set *A* and *A* + *B* with the previous experiments with data set *A* concerning ensembling [7] and artificial neural networks [8].

Ensemble learning consists in learning of single weak and diverse learners [33]. To build an ensemble of models we started from an empty ensemble and added step-by-step the best model in the so called Out-of-Train procedure (OOT) (after Breiman’s Out-Of-Bag technique [34]). Several models were trained on the training set and these models were compared by evaluating the prediction errors on the validation set. The best models became ensemble members until the ensemble got the desired size. Six model families were used for the training: LDA, Kernel Ridge Regression with a polynomial kernel *k*(*x*, *x*′) = (*a* + *x*.*x*′)^*b*^, MLP trained with the RPROP descent (with the changeable number of nodes), Perceptron trained with a second order gradient descent, C4.5 decision trees, and Matlab data trees (dtree).

The wavelet features were learnt by an ensemble of models in this way, that the ensemble optimized (one by one) seven different quality measures: accuracy, precision, F1-score, fp rate, specificity, ber and recall. The final model was tested on a separate unseen set of data and the area under the ROC curve (AUC) was calculated.

In those experiments we measured AUC and its error bar to draw some conclusions about stability of the models. Accuracy and recall were concluded to have the highest AUC levels plus modest error bars. In Fig 18 we plot classification performance of the ensemble built on data set *A* by optimizing the recall quality measure. As we can see there are similar fluctuations of AUC over the wavelet number as in SVM (Fig 11) and the average level of AUC in ensembling is higher than this in SVM by about 6%.

This difference in performance between ensembling and SVM should be attributed both to the different learning paradigms (ensembles may be more ‘flexible’ for optimization as far as high dimensional feature space is concerned), and (less) to the Bayesian optimization process, which is a statistical procedure.

The most optimal wavelet base in our ensembling experiments ([7]) was reported wavelet number 46 i.e. reverse bi-orthogonal wavelet base Rbio3.1 and 26 i.e. bi-orthogonal wavelet base Bior1.5.

The mixture set *A* + *B* (Fig 20) yields similar results, except for more wavelet numbers that satisfy the (almost) constant performance in all the three resolutions i.e. 46, 26, 24, 25, 39. We remember that those wavelet numbers are present in Table 1.

Artificial neural networks (ANN), in turn, is a black-box approach to the knowledge acquisition but can model complex relationships between inputs and outputs due to the nonlinear processing capabilities of its constituent neurons. ANN are widely used as classification systems for the melanoma CAD. In [8] we briefly commented on the existing works on ANN and contributed to the automatic classification of melanoma from dermoscopy images by ANN.

In our study on data set *A* we used 252 input neurons that represented the same set of wavelet features as in this work, extracted for one wavelet base, which was RBio3.1 (wavelet number = 46).

There are no no methodical studies in the literature how the ANN structure (hidden layers) affect the melanoma classification performance. After careful considerations and computational attempts on both CPU- and GPU-based parallel computing platforms we arbitrarily limited ourselves to a few combinations of hidden neurons grouped into one or two hidden layers: [10], [20], [10-10], [20-10], [20-20], which is not a considerably worse setup compared to the literature. The validation data was used to optimize (by mean square error) and update the weights in the backpropagation phase where all the layers had sigmoid activation functions. The testing set was used to calculate AUC on the unknown subset of dermoscopy images from data set *A*.

We used several training algorithms with ‘standard’ base parameters: Levenberg-Marquardt *μ* = 0.001 (L1), Bayesian Regularization *μ* = 0.005 (L2), Broyden-Fletcher-Goldfarb-Shanno (L3), Conjugate Gradient with Powell-Beale restarts (L4), Fletcher-Powell Conjugate Gradient (L5), Polak-Ribiére Conjugate Gradient (L6), Gradient Descent *learn*_*rate* = 0.01 (L7), Gradient Descent with Adaptive Learning *learn*_*rate* = 0.01 (L8), Gradient Descent with Momentum *learn*_*rate* = 0.01, *momentum* = 0.9 (L9), Variable Learning Rate Gradient Descent *learn*_*rate* = 0.01, *momentum* = 0.9 (LA), One Step Secant (LB), Resilient backpropagation *learn*_*rate* = 0.01, Δ = 0.07 (LC), and Scaled Conjugate Gradient (LD).

Our objective was to find the best performing ANN for the classification of melanoma dermoscopy images under the assumption that only those aforementioned limited topologies are taken into account (starting with [10] hidden neuron on one hidden layer up to [20 20] hidden neurons on two layers).

Beside classification performance our experiments had to show the computational efficiency (in terms of learning epochs required to gain a defined error level). For that we grouped results of our ANN setups in terms of pairs (*number*_*ofepochs*, *setups*_*finished*). As a necessary efficiency condition for further analysis we took the median of the number of epochs for the finished setups, which was 20 epochs.

In Fig 21 we plot numerical results of AUC for the resolutions 2272x1704, 1136x852, 568x426, for the five setups of the hidden layers and for the thirteen different back-propagation learning algorithms assumed that the number of epochs is below 20. Absence of L7-LA proves that methods based on (variations of) gradient decent converges very slowly (maximum number of epochs was sometimes even above 1000). Among the remaining cases there are no single winners in our optimization space, different combinations of the learning algorithms and topologies reach high AUC values. Each topology has a preferable learning algorithm assumed a resolution, and in reverse each learning algorithm has the most efficient topology. In any case one can optimally select a triple (*resolution*, *learning*_*alg*, *hidden*_*layers*) to reach a high AUC and match the experimental setup.

In search for resolution invariance we scan through all the resolutions and see that especially L1 (Levenberg-Marquardt) shows extreme robustness for almost all the topologies e.g. (*A*, *L*1, [10 10]) = 0.97, (*A*2, *L*1, [20]) = 1.0, and (*A*4, *L*1, [20]) = 0.98. L6 (Polak-Ribiére Conjugate Gradient) proves to be the second best (*A*, *L*6, [10 10]) = 0.96, (*A*2, *L*6, [20 10]) = 0.99, and (*A*4, *L*6, [10]) = 0.93.

The map of high AUC levels through all the learning functions shows us that images of 1136x852 pixels are the most suitable for classification with ANN (for SVM the preferable resolution was 568x426).

Higher absolute results (although more sparse in the optimization space) are reached for data set *B* (Fig 22). The same applies to Fig 23 for the joint data set *A* + *B*.

Our ANN experiments for one particular wavelet base (wavelet number 46) agree qualitatively with the SVM results and the ensembling results in high classification efficiency and that the wavelet feature (46) is resolution-proof.

Unfortunately no more wavelet bases were studied with ANN due to considerable computational burden.

### Ad.4

A detailed/comprehensive comparison between various methods on the classification of melanoma and benign lesions cannot be done. While the reviews show the techniques and results of the published works, they cannot explain why and to which extent the classification performance varies with the data set, and with the method (the pre-processing step, feature extraction and selection included). This is because not enough information about all the crucial steps of the data collection and analysis are available from the papers, which was criticized by the reviews’ authors. Sometimes even the presented methods have apparent drawbacks.

In fact most of the results falling to the same machine learning paradigm seem to be comparable. This is because we usually compare some absolute performance measures i.e. levels (assumed the same measures) with no variance/dispersion of the results. There are only few works applying a method or the whole pattern recognition system to more than one data set. When different features are extracted/used, comparing performance of even identical machine learning methods is controversial.

There is a lot of melanoma decision support systems, each having its own paradigm, a feature set, and machine learning approaches. Here we want to show our results against the background of existing experiments, based on the review of Masood [3], and more contemporary Oliveira [4] (crucial bibliography can be found there).

Image acqusition, pre-processing, segmentation, feature extraction (and selection), and finally classification are fundamental steps in the non-invasive computer diagnosis of melanoma. Dermoscopic images, we use in this work, are the predominant part of the existing visual material recording skin lesions. This technique was briefly characterized in the introduction. The most important factor that makes this technique most useful in the clinical diagnosis is illumination and magnification of the moles. There are also drawbacks because artefacts such as hair or light reflections and liquid bubbles get magnified as well. For all that dermoscopy remains the most sensitive visual examinations that outperforms plain macroscopic images. Some more advanced techniques like trans-illumination, laser-based methods, ultrasonography or spectral systems are not widely used and are not significant in the mass melanoma screening.

Melanoma image recognition can be reliable when trained on robust and comprehensive data. Unfortunately the only few existing standard data sets that can be used for training and testing in various melanoma CAD systems are relatively fresh and even if some statistics amount to some thousands of images, usually only a fraction of cases can be used. This is due to the variety of classes (non-pigment/pigment lesions, dermal/junctional/compound nevi, typical/dysplastic lesions, melanoma/other skin cancer cases etc.). For the melanoma binary classifiers, which have clinical significance, only melanoma and dysplastic cases should be selected.

The most important existing ML experiments for melanoma usually operate on proprietary small data sets where the melanoma class has 20, 40, sometimes up to 100 cases. They are usually unavailable in public and those experiments are not compared against the standard data sets.

Data set A, which we use, is a medium-size data set for melanoma classification, and its advantage is high resolution, which is not the case of most existing data sets. High resolution images allow for experiments with reduced, downgraded resolutions. In this context data set A is unique. To check performance of the trained SVM model one of the aforementioned public data sets was chosen—data set B described in the section ‘Experimental Data’.

Prior to segmentation some pre-processing steps can be done, if required. They can remove artifacts and enhance contrast. As it was explained in Motivation data sets A and B are taken without any methods enhancing image quality.

Segmentation is an important step to extract regions of interest (ROI) in the image. Lesion border detection methods play an important role and this step has its own vast methodology and classification approaches (SVM included). As it is clearly visible from the literature, segmentation is rather unstable method, which can yield quite good results when fined tuned to the image local conditions. In fact even different images from the same data set can have slightly different magnification and/or illumination parameters (not to mention about the different artifacts), different skin complexions etc. so adjustment to one global (data set wide) parameter set is hard.

Feature extraction methods for melanoma discrimination can be based on diverse grounds. They include clinical approaches (based on asymmetry, border, color, diameter, evolution or elevation of features), widely known as ABCD(E), 7-Point Checklist, Menzies etc., pattern analysis methods, where specific global or local patterns are visible in the dermoscopy image (homogeneous, starburst, globular, etc.), or various shape or color representation/variation features. Finally texture based features may be used, which determine some statistical texture descriptors e.g. the gray-level co-occurence matrix, (dis)similarity, entropy, momenta etc.

Some popular methods to extract those aforementioned features is: thresholding, color discrimination, discontinuity-based segmentation (active contour), region-based segmentation (split and merge, morphological flooding), soft computing and fuzzy logic included.

Other feature extraction methods found in the literature include filter-based approaches: Gabor (dyadic) filters, Fourier power spectrum, Gaussian derivative kernels or multichannel filtering.

Some authors raise objections that a lot of different features are used to feed sophisticated classifiers of melanoma, but there is little discussion about the real meaning of those features and most of the studies do not report the details of their feature extraction procedures.

Wavelet features which are exploited in this work probe the whole area of the image in various scales and frequency subsets, and are sensitive filters of localized frequencies in the skin texture. When this filtering method is done recursively through all the decomposition branches, the wavelet packet transform (WPT) is applied. Identification of malignant melanoma by wavelet analysis is less artifacts sensitive, independent of ‘visual’ conditions of the image thus less error-prone. References on experiments with the wavelet features are collected in the Introduction plus a brief summary is done in [3] and [4]. The wavelets features eliminate the segmentation step so are a promising alternative to most of the feature sets on the market for the melanoma detection. Results of our previous experiments and this work show that selected wavelet features are classification efficient and demonstrate robustness in a chosen spectrum of classifiers (ensembling, ANN, SVM).

Feature selection is used to eliminate redundant, irrelevant or noisy features in order to reduce classifier complexity for better generalization and to make the learning process more time-, memory-, and storage-effective. From the known studies very few report the details of their feature selection procedures. The available ones treat feature selection as an optimization problem and apply greedy, heuristic, or genetic algorithms or some customized approaches. In our method we do not use any feature selection procedures, which was explained in the Motivation.

The classification step to discriminate melanoma lesions vs. dysplastic or other lesions include different learning paradigms. This is usually divided into families of learning methods. They are: artificial neural networks (MLP, RBF), decision trees (CART, C4.5, etc.), Bayesyan networks, support vector machines (linear, polynomial and gaussian kernel) and ensembles (random forest, bagging, boosting, etc.). All those approaches are widely represented in the literature and summerized in the reviews. Below we restrict to SVM-based results.

A brief theory to SVM methods were presented in the Introduction. This is a linear classifier, which can adapt to non-linearity of the model through kernel functions. Despite their inherent advantages, there exist relatively few studies investigating the utility of SVMs in melanoma recognition. SVMs are used as melanoma classifiers in two scenarios:

- as an indirect classifier identifying one of the clinical (dermoscopic) features of melanoma (dots, streaks etc.), thus a feature extraction method, and
- as a global image classifier based on various melanoma features (geometry, colors, wavelet filters etc.).

The first function of SVM is represented in the five main articles.

Celebi et al. [21] took a methodological approach and first determined the lesion border, then they extracted lesion shape and color/texture features. The feature set was optimized by various feature selection algorithms and the optimal feature subset size was ranked by an SVM classifier with the RBF kernel. Experiments on 564 images (15.6% melanoma, 84.4% benign) yielded a specificity of 92.34% and a sensitivity of 93.33% with a feature set of 18 out of all 437 features. The appropriate values of the kernel parameters, C (cost/penalty) and gamma (kernel width), were determined on a grid (*C* ∈ [2^−5^, 2^15^], and *γ* ∈ [2^−15^, 2^3^]). After the grid-search, the SVM classifier was trained with the optimal parameters (*C*, *γ*) = (2.0, 0.125).

In [35] texture, border-based, and geometrical features were extracted from 289 dermoscopy images (114 malignant, 175 benign). The texture features were derived from a wavelet tranform, the border features were derived from spatial and frequency factors of the lesion border model, and the geometry features are derived from shape indexes. The most optimized features were selected by the gain-ratio method and the SVM classifier with the RGB kernel and optimized by SMO. Apart from SVM also random forest, logistic regression, and hidden naive Bayes models were tested. For the 23 features accuracy was 91.26% and ROC was 0.937. The authors concluded that the texture with (fewer) border and geometry features outperform pure texture information only.

Based on color symmetry and texture analysis Abbas et al. [36] developed a system classifying the melanoma tumor patterns (reticular, globular, cobblestone, homogeneous, starburst, parallel ridge, multicomponent pattern). Such multicomponent patterns are well analyzed by learning algorithms that can assign each input pattern to multiple class labels simultaneously. In this work ML-SVM (RBF kernel), ML-kNN and multi-label ranking (AdaBoost.MC) were used, the latter being the best classifier for the problem. For the ML-SVM they reported classification sensitivity of 89.28%, specificity of 93.75% and AUC of 98.6% (as quoted by [4]) and concluded that the developed pattern classifier based on color–texture features agrees with dermatologists’ perception).

Mirzaalian [37] investigated visual streaks as one of the most important dermoscopic criteria for the diagnosis of malignant melanoma. The streaks in 99 dermoscopic images were identified by quaternion tubularness, which is sensitive to the radial components of streaks. Presence or absence of the regular and irregular streaks (measured by some flux-based descriptors for different number of bands, *K* ∈ [5, 13], and thicknesses Δ ∈ [2, 8]) was validated by the SVM classifier (RBF kernel), yielding AUC of 93% in the best model.

Maglogiannis [38] was segmenting and counting dark dots and globules from dermoscopy images (108 benign + 100 melanoma lesions, 632 × 387 pixels) based on inverse non-linear diffusion. The optimal set of the dot features plus some region-based descriptors were classified (among others) by SVM with a polynomial kernel (degree = 5) in the malignant-non malignant lesion setup. For the best feature subset they achieved sensitivity of 88.46%, specificity of 92.31% and accuracy of 90.38%.

The SVM classifier was the main supervised discriminator in the works mentioned below. They are widely commented in the reviews [3, 32, 39].

In [39] the data set contained 1619 lesion images: 600 common nevi, 144 dysplastic nevi, and 65 melanoma for the training set, and 690 common nevi, 80 dysplastic nevi, and 40 melanoma for the test set, all in resolution of 752x582 pixels. The authors analyzed basic, shape and color features (alltogether 107 features) with different normalization conditions and concluded that on both dichotomous (common nevi vs. dysplastic nevi + melanoma; melnoma vs. common + displastic nevi) and trichotomous tasks (correctly distinguishing all three classes) the ANNs performed similarly as logistic regression and SVMs, and better than the k-nearest neighbors and decision trees. For the dichotomous classification the optimal polynomial kernel was linear (*C* = 100) and yielded AUC of 0.92, and the optimal Gaussian RB kernel had inverse variance *γ* = 10^−4^ and *C* = 100 and yielded AUC of 0.97.

[32] reviewed the state of the art of the visual features used for skin lesion classification in the stages: segmentation, border detection and color/texture processing. They also compared the performance of several classifiers and concluded that the SVM classifier (RBF kernel) seems to achieve higher performance in terms of sensitivity and specificity, followed by ADWAT and CART algorithms.

Work [3] is a comparative assessment of skin cancer diagnostic models. They critically examined practices, problems, deficiencies and prospects from the image acquisition to classification of dermoscopic images. Some techniques are commented on the conditions that affect their feasibility.

Single works that are referred to in the above mentioned reviews are briefly commented on below.

Amico et al. [40] constructed various asymmetry measures (the so called Size Functions) to discriminate melanocytic lesions by SVM (kernel function was a third degree polynomial), which was implemented at a clinical level. They gained in the best tests sensitivity of 96.8% and specificity of 87.2%.

[41] examined the melanoma discrimination capacity of some skin specialists (31 melanomas + 103 nevi) and the automatic data analysis for the melanoma early detection system (ADAM, 42 melanomas + 435 nevi). The ADAM system showed a slightly higher sensitivity (84%) and a lower specificity (72%), compared with the physicians.

In [42] a local thresholding algorithm was proposed to extract separation, border, texture and color based features. Those features were used to construct a classifier based on SVM (malignant melanoma versus dysplastic nevus). The best accuracy of the RBF kernel was 91.84% (sensitivity = 91.30%, specificity = 91.87%).

[43] developed a decision support system for the dermoscopic images by combining (by the Bayes theory) outputs from different classifiers (SVM, GML, kNN). On a collection of 358 dermoscopic images they used local color and texture-related features to achieve accuracy of 76%.

In [44] a diagnostic system for dermatologists based on SVM models of melanoma was built. They investigated 14 geometry and color features. They tested four distinct kernels: polynomial, sigmoid, RBF and the k-MOD decreasing. The best SVM model with the k-MOD decreasing kernel function got 89% sensitivity and AUC = 76% using a set of 199 digital dermoscopic images (101 melanomas, 98 dysplastic).

Work [45] aimed at determining the best system for the skin lesion classification out of one global (one of them was SVM, RBF kernel) and one bag-of-features classifier based on local features. The other objective was to compare the role of color and texture features in lesion classification and determine which set of features is more discriminative. They concluded that color features outperform texture features when used alone and that both methods achieve very good results on 176 dermoscopy images, i.e. sensitivity of 92% and specificity of 72% for the SVM global method against sensitivity = 100% and specificity = 75% for local methods.

A classification system for four types of skin cancers: melanoma, basal cell carcinoma (BCC), actinic keratosis (AK), squamous cell carcinoma (SCC) was reported in [46]. The GLCM based texture features ware extracted from each of the four classes and given as input to a multi-class SVM. The accuracy of classification to the one of the four skin cancer classes was 81.43%.

Masood et al. [47] analyzed 168 images (112 melanoma and 56 benign) rescaled to a resolution of 720x472. The lesion area was segmented using the Histogram based Fuzzy C Mean algorithm for Level Set initialization (H-FCM-LS). 45 features (15 GLCM, 5 GTDM-Gray Tone Difference Matrix, 15 FMI-WPT, and 10 Autoregressive) were extracted for each image The FMI-WPT features (Fuzzy Mutual Information based Wavelet Packet Transform (FMI-WPT) were extracted to level 3 and then some fuzzy sets were constructed. The number of features that maximized the classification performance was evaluated using fuzzy-set entropy based criterions. The constructed feature sets are used separately as well as in different combinations for feeding the classifier. Finally, Self-advisable Support Vector Machine (SA-SVM) was used for classification. SA-VM (both linear and kernel based) uses information generated from misclassified data in the training phase and thus, improves performance by transferring more information from training phase to the test phase. The results obtained by SA-SVM were significantly better than the results of traditional SVM. The SA-SVM diagnostic system achieved an overall accuracy of 90%, with sensitivity 91% and specificity 89%.

In [49] features based on asymmetry, border irregularity, color variations and diameter were calculated from an illumination compensated segmented image after noise removal by iterative dilation. The SVM classifier was optimized by Sequential Minimal Optimization (SMO) for the parameters that varied in the ranges: *C* ∈ 0.1, 0.2, …, 5 for soft margin and *σ* ∈ [1, 11]. The achieved sensitivity and specificity for the different sets of training and test data elements were respectively 87% and 94% (1-fold), and 90% and 75% (10-fold CV).

Amerald et al. [50] proposed the high-level intuitive features (HLIFs) to model the ABCD criteria commonly used by dermatologists. They experimented with various data sets concluding that concatenating the proposed HLIFs with some low-level features increased classification accuracy. The SVM classifier with the linear kernel on the best data set achieved sensitivity 92.52%, specificity 96.22%, and accuracy 96.64%.

Choudhury et al. [51] proposed a multilayer decomposition based textural and color features for SCC, BCC, melanoma and actinic keratosis. The normalized GLCM and histogram of oriented gradients (HOG) were used as textural feature descriptors, and were extracted from different layers of the image than color histograms. The base and detailed layers were decomposed by the weighted least squares (WLS) edge-preserving decomposition. These features were fed to multi-class SVM (MSVM) and extreme learning machine (ELM) for classification. An average accuracy of 94.18% for MSVM was better than 90.5% with ELM.

Alquran et al. [52] collected asymmetry, border, color and diameter (ABCD) features extracted using the GLCM method after segmentation using thresholding. Those features were selected by principal component analysis (PCA), than the Total Dermoscopy Score (TDS)then the SVM classification was done. The achieved classification accuracy was 92.1%.

The most recent work on SVM classified malicious and benign skin lesions by Ashtami [53] proposed new features characterizing border irregularities on both complete and incomplete lesions. Those features plus color and texture features (GLCM) were classified by a Support Vector Machine (SVM) model on two dermoscopy databases with images of two human races: caucasian an xanthous race. Their results, sensitivity 97.82%, specificity 75%, and accuracy 88.46% are slightly better than for their ANN classifier.

There are few direct SVM systems based on wavelet features (and they are not fully comparable with our experiments):

Surowka et al. [14] tested performance of several machine learning paradigms and methods: neural networks, support vector machines, and Attributional Calculus, applied to dermatoscopic images of potentially malignant pigmented lesions. The features were obtained using the multiresolution wavelet-based decomposition of the image. The SVM AUC was 0.937.

In [48] 255 features were extracted from Wavelet Packet Transform (WPT) and reduced by Particle Swarm Optimization (PSO) to optimize the SVM classifier. In the pre-processing step the Wiener2, Gabor, median filtering and histogram equalization was introduced to the images. Each image was further segmented by edge detection and thresholding. All the tasks yielded classification sensitivity of 94.1%, specificity of 80.22%, and accuracy of 87.13%

Takruri et al. [16] analyzed 448 digital and dermoscopic images (benign and malignant) from two sources and segmented them by k-means clustering. They derived wavelet (db4), curvelet and color based features both from grayscale and color original images which resulted in a sensitivity of 86.4% and 76.9% and specificity of 88.1% and 85.4% for the wavelets and curvelets respectively. The obtained results were discussed to be comparable to those obtained by dermatologists.

In Table 2 we show our SVM numerical results (best wavelet numbers in each data set plus the parameters) against all the quoted literature results (Tables 3 and 4) for the SVM learning paradigm of non-wavelet and wavelet features. We conclude that our results are reliable and compatible to the rest of the works. Unfortunately, due to too few melanoma classification systems utilizing both the wavelet features and the SVM classifiers plus unique conditions of the existing experiments, and last but not least, lack of details from the groups, makes a full quantitative comparison still unfeasible.

**Table 2:** Results of the melanoma SVM learning (this work). Legend: dn = dysplastic nevus, cn = common nevus. RBF kernel parameter: *γ*, polynomial kernel parameter: degree; #features = 252, partition: 10-fold CV. Resolutions: *A* = *A* + *B* = 2272 x 1704, *B* = 760 x 552, *A*2 = *A*2 + *B*2 = 1136 x 852, *A*4 = *A*4 + *B*4 = 568 x 426.

Data set	#images	#class A (melanoma)	#class B	wavelet number	wavelet base	kernel param.	C	AUC	#supp.vect.
gaussian									
A	185	102	83 dn	46	Rbio3.1	4.68e-09	9.81e+06	0.870	72
A2	185	102	83 dn	26	Bior1.5	2.41e-04	9.69e+06	0.859	81
A4	185	102	83 dn	46	Rbio3.1	3.72e-06	4.76e+04	0.859	60
B	113	40	73 (cn + dn)	3	Db2	1.61e-03	1.54e+06	0.932	68
A + B	298	142	156 (cn + dn)	41	Rbio1.5	6.98e-04	6.96e+02	0.881	120
A2 + B2	298	142	156 (cn + dn)	24	Bior1.1	4.25e-03	2.36e+04	0.877	177
A4 + B4	298	142	156 (cn + dn)	26	Bior1.5	5.03e-03	1.64e+04	0.854	218
polynomial									
A	185	102	83 dn	48	Rbio3.5	3	9.53e+06	0.870	73
A2	185	102	83 dn	26	Bior1.5	3	5.78e+05	0.854	83
A4	185	102	83 dn	26	Bior1.5	4	6.50e+05	0.859	102
B	113	40	73 (cn + dn)	26	Bior1.5	3	1.53e+00	0.940	50
A + B	298	142	156 (cn + dn)	46	Rbio3.1	6	7.21e+03	0.868	92
A2 + B2	298	142	156 (cn + dn)	2	Db1	4	2.56e+05	0.871	125
A4 + B4	298	142	156 (cn + dn)	46	Rbio3.1	2	2.33e-01	0.868	138
linear									
A	185	102	83 dn	46	Rbio3.1	-	2.63e+04	0.870	73
A2	185	102	83 dn	46	Rbio3.1	-	5.48e+01	0.843	76
A4	185	102	83 dn	46	Rbio3.1	-	8.28e+03	0.854	62
B	113	40	73 (cn + dn)	40	Rbio1.3	-	5.58e+00	0.923	41
A + B	298	142	156 (cn + dn)	41	Rbio1.5	-	9.39e+06	0.871	106
A2 + B2	298	142	156 (cn + dn)	47	Rbio3.3	-	1.09e-05	0.858	118
A4 + B4	298	142	156 (cn + dn)	46	Rbio3.1	-	1.16e-05	0.854	123

**Table 3:** The SVM-based melanoma machine learning experiments. Legend: n = nevus, cn = common nevus, dn = dysplastic nevus, otherwise = benign; parameters for the RBF kernel: (*C*, *γ*); parameter for the polynomial kernel: (degree).?/blank space = data unavailable.

Ref (resolution)	#images	#class A melanoma	#class B	#class C	partition	#features (best)	kernel (params)	sens	spec	acc	AUC
[39] (752x582)	1619	65/40	600/690 cn	144/80 dn	train/test	107	poly (1) RBF (100, 10^−4^)	84,5% 92,1%	88,5% 95%		92% 97%
[40] (magnif 16x)	977	25/25	500/427 n		train/test		poly (3)	96,4%	87,2%		
[41] (magnif 16x)	477	22/20/ 31 of 42	218/217/ 103 of 435		train/test/ test doctors			84%	72%		
[42]	1041	69	972 n				RBF (?, 7)	91.3%	91.9%	91.9%	
[14] (2272x1704)	39	19	20 dn			231(10)	RBF (512, 0.000244)				97.4%
[21]	564	88	476		10-fold CV	437(18)	RBF (2, 0.125)	93.3%	92.3%		
[43]	358	134	106	118 dn			RBF			75.7%	
[44]	199	101	98 dn		130train/ 69test	14		89%	64%		76%
[35]	289	114	175			(23)	RBF			91.3%	93.7%
[37] (768x512)	99						RBF				93%
[45]	176						RBF	92%	72%		
[36] (768x512)	350				80/20% train/test		RBF (1,?)	89.3%	93.8%		98.6%

**Table 4:** The SVM-based melanoma machine learning experiments (contd). Legend: dn = dysplastic nevus, otherwise = benign; parameters for the RBF kernel: (*C*, *γ*); BCC = Basal Cell Carcinoma, SCC = Squamos Cell Carcinoma, a.ker. = actinis keratosis. blank space = data unavailable.

Ref (resolution)	#images	#class A melanoma	#class B	#class C	partition	#features (best)	kernel (params)	sens	spec	acc	AUC
[38] (632×387)	208	100	108				poly (5)	88.5%	92.3%	90.4%	
[46] (150x112)	359	77	BCC(84) a.ker.(101) SCC(101)							81.4%	
[47] (720x472)	168	112	56		84 train 84 test	45		91%	89%	90%	
[48]	79	50	29		64 train/ 15 test 15 test	255	RBF (3525.0051, 0.0084732)	94.1%	80.2%	87.1%	
[16]	448	93 + 142	121 + 92		0.8train/ 0.2test	52 55(25)	RBF	86.4% 76.9%	88.1% 85.4%		
[49]	146	108	38		1-fold CV 10-fold CV		RBF	87% 90%	94% 75%		
[50]					LOO		linear	92.5%	96.2%	96.6%	
[51]										94.2%	
[52]										92.1%	
[53]						151		97.8%	75%	88.5%	

## Conclusion

Computer aided diagnostic systems are common. For the early detection of cutaneous melanoma they play a crucial role to support clinics and general practitioners. Our work contributes to this effort in the field of feature extraction. Appropriate features project the information from a (dermoscopy) image into a space where classes (malignant and benign lesions) are well separated. This step is critical for the performance of the classifier and the learning procedure.

In this work we extracted features from the WPT wavelet transform for the sake of efficient SVM classification. We analyzed 53 wavelet bases to select those that are efficient for the SVM learning, and to probe the SVM parameter space. Our experiments were performed on two different data sets of unequal resolution, and biased with different illumination, representation, and compression.

The SVM paradigm involves optimization of a convex cost function, therefore it is a ‘global’ classifier, less prone to overfitting. It is flexible in a high dimension feature space through an appropriate choice of kernel, is robust and simple.

We tested the linear, polynomial and gaussian kernel and searched (Bayesian optimization) the kernel parameters (C, *γ*, degree of polynomial). From this analysis we concluded that:

1. Kernel functions play a key role in the performance of the SVM classification, the best kernel was the gaussian function.
2. Wavelet features classified by SVM with different kernel functions have ‘preferential’ resolutions where they reach the most optimal classification efficiency. Analysis on two data sets (A, B) shows that for the gaussian kernel such most efficient resolution is 568x426. For the melanoma classification lower image resolution would be potentially resource-saving without (noticeable) degradation of the classification performance.
3. We selected wavelet bases that performed the best for a certain resolution and SVM kernel function, and also such that keep high classification efficiency towards downgraded image resolutions (Rbio and Bior wavelet families, particularly Rbio3.1 and Bior1.5).

Our findings are compatible with our previous machine learning experiments on the melanoma discrimination (ensembling and artificial neural networks) where high classification accuracy and stable behavior over a range of resolutions was observed as well.

Regarding our SVM classification of melanoma against dysplastic nevus, our classification performance is in line with other research groups, however a detailed comparison with other experiments with different data sets, selection criteria, different pre-processing steps and finally different features is problematic.

General outlook is that further analyses on various (large) public data sets of dermoscopic skin lesion images should be done to probe new learning paradigms and yield quantitative results contributing to the melanoma feature extraction.
